# Understanding Xeroderma Pigmentosum Complementation Groups Using Gene Expression Profiling after UV-Light Exposure

**DOI:** 10.3390/ijms160715985

**Published:** 2015-07-14

**Authors:** Nikola A. Bowden, Natalie J. Beveridge, Katie A. Ashton, Katherine J. Baines, Rodney J. Scott

**Affiliations:** 1 Hunter Medical Research Institute and the University of Newcastle, Callaghan, NSW 2289, Australia; E-Mails: natalie.beveridge@newcastle.edu.au (N.J.B.); katie.ashton@newcastle.edu.au (K.A.A.); katherine.baines@newcastle.edu.au (K.J.B.); rodney.scott@newcastle.edu.au (R.J.S.); 2 Hunter Area Pathology Service, Pathology NORTH, New Lambton Heights, NSW 2305, Australia

**Keywords:** xeroderma pigmentosum, transcriptome, gene expression profile, UV, nucleotide excision repair

## Abstract

Children with the recessive genetic disorder Xeroderma Pigmentosum (XP) have extreme sensitivity to UV-light, a 10,000-fold increase in skin cancers from age 2 and rarely live beyond 30 years. There are seven genetic subgroups of XP, which are all resultant of pathogenic mutations in genes in the nucleotide excision repair (NER) pathway and a XP variant resultant of a mutation in translesion synthesis, POLH. The clinical symptoms and severity of the disease is varied across the subgroups, which does not correlate with the functional position of the affected protein in the NER pathway. The aim of this study was to further understand the biology of XP subgroups, particularly those that manifest with neurological symptoms. Whole genome gene expression profiling of fibroblasts from each XP complementation group was assessed before and after UV-light exposure. The biological pathways with altered gene expression after UV-light exposure were distinct for each subtype and contained oncogenic related functions such as perturbation of cell cycle, apoptosis, proliferation and differentiation. Patients from the subgroups XP-B and XP-F were the only subgroups to have transcripts associated with neuronal activity altered after UV-light exposure. This study will assist in furthering our understanding of the different subtypes of XP which will lead to better diagnosis, treatment and management of the disease.

## 1. Introduction

The childhood skin cancer disorder Xeroderma Pigmentosum (XP), and the childhood neurological disorders Cockayne’s syndrome (CS) and trichothyodystrophy (TTD) all occur as a result of autosomal recessive mutations in 1 of 10 genes involved in the nucleotide excision repair (NER) pathway [1]. The three diseases are not mutually exclusive, some children present with symptoms of XP and CS or XP and TTD [2,3,4]. Children with XP have severe UV-light sensitivity as a result of diminished DNA repair activity [5]. NER is responsible for removing thymine-dimers and bulky DNA adducts including the carcinogenic cyclobutane pyrimidine dimers induced by UV irradiation [6].

UV-light induced DNA damage both distorts and covalently modifies DNA, requiring the precise NER DNA repair process to correct the damage. In XP, absence of functional NER results in up to 2000 times greater susceptibility to uniformly distributed melanomas and 10,000-fold increase in basal cell carcinomas (BCCs) and squamous cell carcinomas (SCCs) [7]. The first presentation of skin cancer in XP is usually at approximately 2 years of age [8]. XP has a worldwide population frequency of approximately 1 in 1 million [9] but has a higher frequency in Japan [10] and Mediterranean areas [11] and affected children have a life expectancy of 10 to 30 years. CS is diagnosed by the presence of developmental and neurological symptoms, but can manifest with the severe UV-sensitivity of XP, therefore some children have a combination of XP/CS. TTD is the most rare of the NER disorders and is characterised by clinical features such as brittle hair and ichythiosis. Similar to CS, TTD can manifest with XP to produce a child with a combination of XP/TTD [2,3,4]. (Outlined in Table 1).

XP can be further sub-divided into seven distinct subgroups, known as complementation groups, XP-A through to XP-G as well as a milder variant form known as XP-variant (XP-V). Each complementation group refers to the presence of a causative mutation in one of the seven XP genes involved in NER [12,13] or POLH which is involved in translesion systhesis [14].

DNA damage recognition occurs via global genome repair (GGR) or transcription coupled repair (TCR). After damage recognition occurs, the remainder of the NER pathway removes and repairs the DNA lesion. As the name suggests, TCR detect DNA damage during transcription when RNA polymerase II stalls at a site of damage and recruits CSA and CSB. GGR is responsible for damage detection across the remainder of the genome and involves XPC and the DDB1/DDB2 complex. After the DNA damage is detected via GGR or TCR the remainder of the XP proteins are involved in DNA unwinding (XPA, XPG, XPB, XPD) and excision of the damage (XPF, ERCC1, XPG). The location of the affected gene in the NER pathway does not confer severity of DNA repair deficiency and subsequent clinical manifestation of the disease. Therefore, accurate diagnosis of XP, CS and TTD currently relies on a combination of clinical assessment and identification of mutations in the NER genes.

**Table 1:** Genes involved in xeroderma pigmentosum, Cockayne’s syndrome and trichothiodystrophy.

Gene	Genomic Location/Size	Coding Sequence Size (bp)	Clinical Manifestation	UV-Sensitivity	Residual DNA Repair	Relative Frequency	Skin Cancer	Neurological Implications
Global Genome Repair								
*XPC*	3p25	3558	XP	++	<10%–30%	High	+	−
*DDB1*	11q12–q13	3420	N/A	N/A	N/A	N/A	N/A	N/A
*DDB2 (XPE)*	11p12–p11	4193	XP	+	50%	Rare	+	−
Transcription Coupled Repair								
*CSA*	5q12.1	2011	CS	+	Normal	Intermediate	−	++
*CSB*	10q11.21	4714	CS	+	Normal	High	−	++
Nucleotide Excision Repair								
*XPA*	9q22.3	1377	XP	+++	<5%	High	+	++
*XPB*	2q14.3	2751	XP, XP/CS, TTD	++	3%–40%	Rare	+/−	+
*XPD*	19q13.3	2400	XP, XP/CS, TTD	++	15%–50%	Intermediate	+	++/−
*XPF*	16p13.1	2881	XP	+	15%–30%	Rare	+	−/+
*XPG*	13q33.1	4091	XP, XP/CS	++	<5%–30%	Rare	+	++
*ERCC1*	19q13.3–q13.2	N/A	N/A	N/A	N/A	N/A	N/A	N/A
*TTD-A*	6p25.3	7503	TTD	N/A		Rare	−	++
Translesion Synthesis								
DN/A PolH (XPV)	6p21.1	2140	XP	−	Normal	High	+/−	−

XP: Xeroderma Pigmentosum; CS, Cockayne’s syndrome; TTD: trichothyodystrophy; N/A = not applicable, + = mild, ++ = moderate, +++ = severe and − = not present.

A potential mechanism to understand the biological differences seen in each XP subtype is whole genome gene expression analysis. This approach has been used successfully for understanding subtypes of common cancers, particularly breast cancer [15]. The aim of this study was to use whole genome gene expression profiling of fibroblast cell lines from individuals with known XP mutations to determine distinct expression patterns associated with each subtype of the disease. UVC irradiation was used to efficiently elicit DNA damage that triggers a DNA repair response via NER, and differential gene expression for each XP complementation group was analysed to identify defining gene expression profiles and biological processes for each XP complementation group.

## 2. Results and Discussion

**Figure 1:**
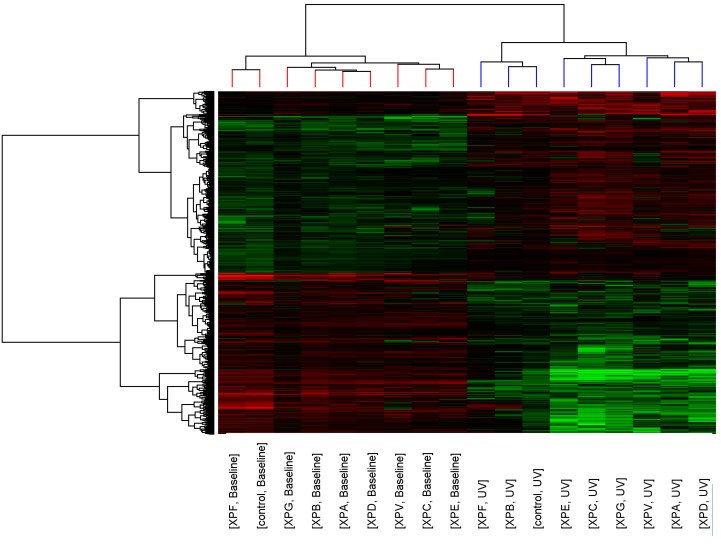
Cluster analysis of control and XP complementation groups before and after UVC-light. Each column represents average transcript expression for duplicate cell lines for control and each of the XP complementation groups before and after 2 J/m^2^ UVC-light exposure. Down-regulation is represented as green, up-regulation as red and normal expression as black. The dendrogram represents the relationship between and within the controls and XP complementation groups.

Whole genome transcript expression data was collected for control fibroblasts and XP-A, XP-B, XP-C, XP-D, XP-E, XP-F, XP-G and XP-V fibroblasts before and after 2 J/m^2^ UVC-light exposure for one minute. To ensure the pattern of altered transcript expression was robust, two separate cell lines were used for control fibroblasts and each XP complementation group with the exception of XP-E and XP-F where only one cell line was available (details in Table 2). The analysis pipeline to determine if distinct profiles for each XP complementation group could be identified was similar to our preliminary study [16]. Briefly, to ascertain the relatedness of each XP complementation group to the control fibroblasts, standard correlation and distance were used for hierarchical clustering to show relationships between expression profiles for the 7913 transcripts with significantly altered expression after UV-light exposure (*p* < 0.05, >2-fold change). Similarity of transcript expression profiles is measured by distance; the vertical height of the branches of the dendrogram (Figure 1), therefore the closer the relatedness of one profile to another the shorter the vertical branch. A second dendrogram (Gene tree) was created using standard correlation and distance to show relationships between the expression levels of transcripts across the groups.

Before UV-light irradiation the cluster pattern of the XP complementation groups showed no relationship to DNA repair capacity or clinical severity of the disease (Figure 1). Three distinct clusters formed consisting of XP-C, XP-E (both form part of global genome repair) and XP-V (translesion synthesis); XP-G, XP-B, XP-A and XP-D (all members of the DNA unwinding and excision complexes of NER); and XP-F and control. After UV-light irradiation the relatedness of the control fibroblasts and XP complementation groups was altered and a distinct cluster containing control, XP-B and XP-F was seen.

**Figure 2:**
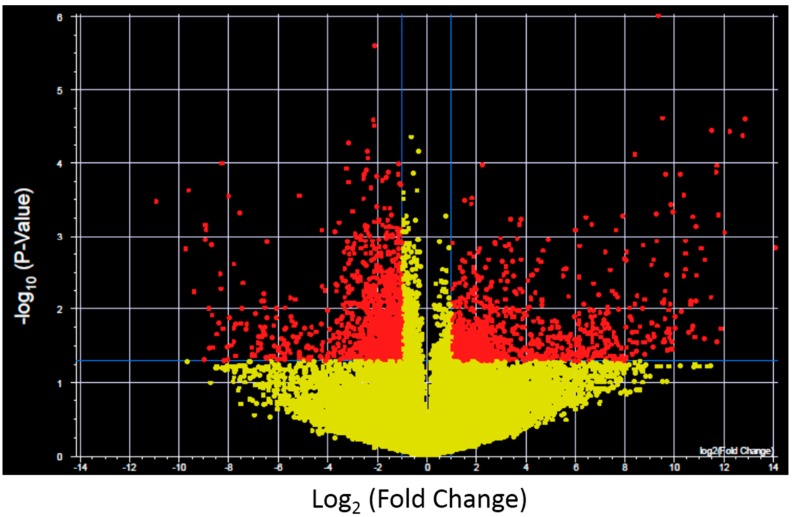
Example of a Volcano plot of altered transcript expression in control fibroblasts after UVC irradiation. −log_10_ (*p*-value) plotted against log_2_ (Fold Change) for transcript expression in control fibroblasts before and after 2 J/m^2^ UVC irradiation. Red spots represent 2575 transcripts with *p* < 0.05 and fold change >2, yellow spots represent transcipts that were not differentially expressed. Volcano plots were generated for each XP complementation group.

**Figure 3:**
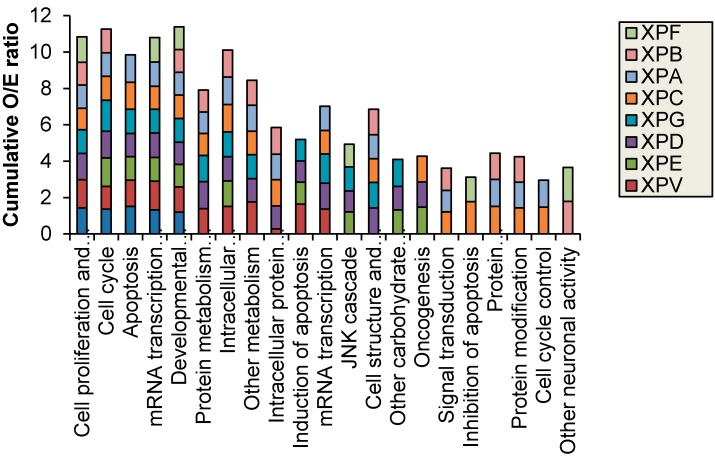
Biological processes with significantly (*p* < 0.05) different observed (O) *vs.* expected (E) ratio of transcripts with altered expression after 2 J/m^2^ UVC-light treatment. XP-B and XP-F were the only XP complementation groups to have altered neuronal function.

To further investigate the expression changes specific for each XP complementation group transcripts with significantly altered expression and >2-fold change after UV-light exposure were identified for control fibroblasts and each XP complementation group using a volcano plot (Figure 2 shows results for control fibroblasts). Significantly altered transcripts representative of biological pathways including cell cycle, inhibition and induction of apoptosis, mRNA transcription and oncogenesis were altered and were identified in both the NER deficient and control fibroblasts (Figure 3). The control fibroblasts had 2576 transcripts with altered expression after UV-light, which was significantly less than the majority of XP complementation groups (Full lists in Supplementary Table S1). This is in contrast to our previous finding where only XP-C and XP-D had higher levels of altered transcripts [16], the difference is lost likely due to the 4-fold increase in the total number of transcripts investigated in the current study. The transcripts altered by UV-light in the control fibroblasts were predominantly involved in cell cycle, apoptosis and mRNA transcription regulation. The XP cell lines also displayed an over-representation of altered protein metabolism, modification and trafficking as well as intracellular signaling. Interestingly, the individuals with XP-B and XP-F formed a separate group in the hierarchical cluster. These XP complementation groups had fewer oncogenic functions/pathways altered after UVC-irradiation and were the only XP complementation group to have an over-representation of significantly altered transcripts with neuronal activity (Figure 3).

The severity of XP and age of diagnosis are mainly dependent on exposure to sunlight, the XP complementation group and the type and functional location of the pathogenic mutation. Despite this, accurate XP complementation group diagnosis is rarely made as it relies solely on identification of the recessive pathogenic mutations present. XP patients without neurological symptoms, who are diagnosed at an early age and carry out stringent UV protection have a relatively good prognosis [17]. Therefore, understanding the biological basis of XP-related neurological abnormalities and quicker, easier and more reliable genetic testing is vital.

Gene expression profiling has successfully been used to find subtypes of disease, with the most reproducible results seen for breast cancer subtypes [15]. To reproduce similar results for XP, we hypothesized the greatest difference between each complementation group would be seen after UV-light exposure when the differing levels of DNA repair defect are apparent. Previous studies have reported high numbers of differentially expressed transcripts in fibroblasts after UV-light exposure compared to other cell types such as cardiac myocytes [18], therefore fibroblasts may be more amenable than other readily accessible cell types when studying the response of DNA repair pathways after UV-light exposure. Our previous study, used gene expression analysis of 6000 transcripts to measure the level of altered gene expression present in six XP fibroblast cell lines after UV exposure and to determine if distinct gene expression profiles exist for each XP complementation group [16]. The study confirmed that distinct gene expression profiles from a portion of the genome, are identifiable for each XP complementation group. Direct comparison between our 2006 study [16] and the current study revealed broad biological processes that were altered in the complementation groups with neurological symptoms (Figure 3). The exact transcripts with altered expression were difficult to compare given the differences in array platforms (dual colour spotted oligonucleotide microarrays *vs*. single-colour Illumina beadarrays), the 4-fold increase in transcripts and the increase to two cell lines for the majority of complementation groups. Nevertheless, the same pattern of distinct changes in the complementation groups with neurological symptoms was present in both studies.

The study reported herein confirms fibroblasts are ideal to study whole genome transcript expression profiles in disorders with reduced NER activity. A high number of transcripts were altered in the control fibroblasts after UV-light exposure and an even higher number of transcripts were altered in most of the NER deficient XP fibroblasts providing a large dataset to use as the basis for determining if each XP complementation group displayed a distinct set of altered transcripts. The control fibroblasts had 2576 transcripts with altered expression after UV-light, which was significantly less than the majority of XP complementation groups (Full lists in Supplementary Table S1). This is most likely due to the genome-wide effect of dysfunctional NER, the less DNA repair activity is present the more effect it will incur across the genome, particularly after a major DNA insult such as UV-light exposure. In this study the UV wavelength used was UVC. Although UVC is completely absorbed by the earth’s atmosphere and as a result UVA and UVB make up the UV-spectrum at the earth’s surface, UVC elicits the greatest mutagenic effect on DNA and in the laboratory setting requires 1000-fold less energy than UVB, therefore UVC was chosen for this study.

The transcripts with altered expression were predominantly involved in cell cycle, apoptosis and mRNA transcription regulation which are all well characterized responses to UV-light irradiation [19,20]. In addition to these biological functions being altered, the XP cell lines also displayed an over-representation of altered protein metabolism, modification and trafficking as well as intracellular signaling. Altered function of these processes may be a downstream effect of DNA repair deficiency. Interestingly, the individuals with XP-B and XP-F formed a separate group in the hierarchical cluster and had absence of UV-sensitivity [21] or late onset of skin malignancies (GM03542 XPF patient first reported skin malignancy at 29 years) and presence of neurological symptoms [1]. XP-B and XP-F were the only XP complementation groups to have an over-representation of significantly altered transcripts with neuronal activity, this is despite neurological symptoms being present in XP-A, XP-D and XP-G patients. The mild clinical manifestation and lack of UV-sensitivity in the XP-B and XP-F patients used in this study may explain the distinct profiles identified. To further expand on this study the altered transcription effects specific to each XP complementation group should be investigated in isogenic XP cells with uncorrected and corrected mutations in future studies.

A recent study reported the long-term follow-up of 106 XP patients admitted to the National Institutes of Health (NIH) from 1971 to 2009 [7]. Progressive neurologic degeneration was present in 24% (*n* = 25) and the median age at death (29 years) in XP patients with neurodegeneration was significantly younger than those without neurodegeneration (37 years) (*p* = 0.02). The underlying biology of the neurodegeneration associated with XP remains to be elucidated but it appears to have a negative impact on the survival of XP patients. In this study, we found that the XP-B and XP-F complementation groups with confirmed neurological symptoms and lack of UV-sensitivity, clustered separately and had very distinct gene expression profiles. Although skin fibroblasts were used, the results indicated the function of neuronal related pathways was significantly affected by UV-light exposure. In the context of XP patients with neurological manifestations, it is likely that non-UV forms of DNA damage such as oxidative damage are accumulating, resulting in neurodegeneration [22]. However, this requires further investigation.

## 3. Experimental Section

Cell lines: Control and XP fibroblast cell lines were obtained from the NIGMS Human Genetic Cell Repository, Coriell Institute for Medical Research, the details of the cell lines are summarized in Table 2. Clinical information was collated from the NIGMS Human Genetic Cell Repository and as previously reported [1,23]. Duplicate healthy control fibroblasts and duplicate fibroblast cell lines from each XP complementation group, with the exception of XP-E and XP-F, were used for the study. All XP cell lines had previously identified pathogenic mutations with the exception of one XP-G and XP-V which were designated by complementation studies (GM03021B) or based on clinical symptoms (GM02004). All fibroblast cell lines were grown in standard conditions (1× complete DMEM, 10% FCS, 37 °C, 5% CO_2_).

UVC-treatments: Each cell line was irradiated with UVC-light (2 J/m^2^ for 1 min) and left to recover (1 h, 1× DMEM 10% FCS, 37 °C, 5% CO_2_) before RNA was extracted for transcript expression analysis.

RNA extraction and microarray procedure: RNA was extracted (Trizol reagent, Invitrogen, Carlsbad, CA, USA) from treated and non-treated cell lines as per manufacturer’s instructions. The RNA was cleaned (RNeasy Kit, Qiagen, Hilden, Germany) and quantified using a fluorometer and RiboGreen reagent (Invitrogen). 500 ng of RNA was amplified and biotinylated using the Ambion Illumina TotalPrep kit (San Diego, CA, USA). After quantification of the amplified RNA, 1–2 µg of biotinylated cRNA was hybridised with a Whole Genome Gene Expression BeadChip (Illumina) containing approximately 24,000 transcripts.

**Table 2:** Clinical characteristics and mutation status of XP and control fibroblast cell lines.

Coriell ID	Complementation Group	Sex	Age	Mutation	Estimated DNA Repair Capacity	UV Sensitivity	Neurological Symptoms	Diagnosed Disorder
GM03652	Control	M	24	N/A	-	No	No	N/A
GM00023A	Control	F	31	N/A	-	No	No	N/A
GM00544B	XP-A	M	10	468–469 del	2%	unknown	unknown	XP with features of CS
GM02009	XP-A	F	10	349–353 del frameshift termination, 323G>T	-	Yes	Yes	XP
GM13025	XP-B	M	39	T>C missense mutation	-	No	Yes	XP with features of CS
GM13026	XP-B	M	36	T>C missense mutation	-	No	Yes	XP with features of CS
GM00030A	XP-C	M	24	83 bp insertion at 462 in cDNA	10%–20%	Yes	No	XP
GM01881B	XP-C	F	45	1132–1133 del resulting in termination	10%–20%	Yes	No	XP
GM00435A	XP-D	F	23	2047C>T cDNA	30%–50%	Yes	Yes	XP with features of CS
GM03248A	XP-D	M	5	1805G>A	30%–50%	Yes	Yes	XP with features of CS
GM01389A	XP-E	F	21	1224T>C missense mutation, del 1220–1222	60%	Yes	No	XP
GM03542C	XP-F	M	29	1790T>C	10%	No	Yes	XP
GM03021B	XP-G	F	17	Not known *	0%	Yes	Yes	XP
GM16180	XP-G	F	-	1116 del TC frameshift truncation	-	Yes	Yes	XP with features of CS
GM02004	XP-V	-	-	Not known	-	unknown	No	XPV

* XPG confirmed by complementation studies [23]; XP: Xeroderma Pigmentosum; CS, Cockayne’s syndrome.

Data analysis: The transcript expression results were initially cubic spline normalised and the expression profiles and each individual transcript were normalized to the median resulting in two-way normalisation. Volcano plots were generated using Welch’s ANOVA and Benjamini and Hochberg false discovery rate (5%) and fold change of greater than 2. PANTHER [24,25] was used to identify biological functions with significantly different (*p* < 0.05) observed (O) number of transcripts compared to expected (E). The O/E ratio was used to indicate the over-representation of specific biological processes rather than absolute number of transcripts. To investigate the relatedness of the XP complementation groups to each other, cluster analysis was performed using Pearson’s correlation and average linkage algorithm for clustering.

## 4. Conclusions

The expression profiles within XP complementation groups appeared to be highly specific and after UV-irradiation, also reflects the clinical symptoms seen in XP. The biological pathways with altered gene expression after UV-light exposure were distinct for each subtype and contained oncogenic related functions such as perturbation of cell cycle, apoptosis, proliferation and differentiation. Patients from the subgroups XP-B and XP-F were the only subgroups to have transcripts associated with neuronal activity altered after UV-light exposure. The results of this study confirm that cluster analysis can identify distinct gene expression patterns for each XP complementation group. The identification of distinct gene expression profiles for XP complementation groups will further our understanding of the biology of XP and may assist in diagnosis of the disease, which currently relies on complementation studies [26]. In addition, the results add to our limited understanding of the biological cause of neurological symptoms in XP, an area that needs further investigation. Understanding the biology associated with pathogenic mutations in XP, CS and/or TTD will allow for better diagnosis, disease management and informed genetic counselling.

## Supplementary Materials

Supplementary materials can be found at http://www.mdpi.com/1422-0067/16/07/15985/s1.
